# The correlation between the costs and clinical benefits of national price-negotiated anticancer drugs for specific cancers in China

**DOI:** 10.7189/jogh.13.04140

**Published:** 2023-11-09

**Authors:** Yuwen Bao, Yanyan Liu, Rui Ma, Pei Zhang, Xin Li

**Affiliations:** 1 Department of Health Policy, School of Health Policy and Management, Nanjing Medical University, Nanjing, China; 2 Department of Organization and Human Resource, Nanjing Drum Tower Hospital, Nanjing, China; 3 Department of Pharmaceutical Regulatory Science and Pharmacoeconomics, School of Pharmacy, Nanjing Medical University, Nanjing, China; 4 Center for Global Health, School of Public Health, Nanjing Medical University, Nanjing, China

## Abstract

**Background:**

The high costs of novel anticancer drugs have caused concern among healthcare stakeholders. To address the knowledge gap on the proportion of survival benefit with the related economic expenditure, we aimed to assess the correlation between the costs and value of innovative drugs targeted to specific tumours, before and after price negotiation policy implementation.

**Methods:**

We identified new drugs for lung and breast cancer that entered the National Reimbursement Drug List (NRDL) through price negotiation from 2016 to 2023. Therapeutic value consisted of traditional clinical endpoints, like the percentage improvement of overall survival (ΔOS%) and progression-free survival (ΔPFS%), and the quantified gains of the American Society of Clinical Oncology Value Framework (ASCO-VF) and the European Society for Medical Oncology Magnitude of Clinical Benefit Scale (ESMO-MCBS). We calculated monthly drug costs and used Spearman’s correlation coefficient and Cohen’s kappa statistics for statistical analysis.

**Results:**

Twenty-nine innovative price-negotiated drugs were collected between 2016 and 2023. The median monthly costs were US$3381.31 out of NRDL and US$1095.88 within NRDL, with an ΔOS% of 22.24% (IQR = 6.45-29.48) and a ΔPFS% of 83.82% (IQR = 50.41-104.05). The median ASCO-VF score was 40.98, and 17 drugs scored the meaningful benefit of ESMO-MCBS. We found no association between clinical benefits and their costs before and after NRDL, either overall or for specific cancers. The agreement between the two frameworks was stable.

**Conclusions:**

The negotiation policy decreased medication costs, but did not generate the expected correlation between the value and costs of anticancer drugs. Comprehensive value assessments need to be performed in the future to explore more in-depth findings and promote the affordability and availability of effective anticancer drugs.

Cancer is a major global health issue and the leading cause of death in China [1,2]. According to the GLOBOCAN 2020 report, an estimated 19.3 million new cancer cases and nearly 10.0 million cancer deaths occurred worldwide in 2020 [3]. China accounted for about 24% (4.6 million) of new cases and 30% (3.0 million) deaths globally [4], indicating an immense cancer burden on patients and the public health system. Meanwhile, the emergence of innovative anticancer therapies has significantly mitigated this health threat and improved future prognosis [5], potentially increasing survival benefits, alleviating cancer pain, and enhancing the quality of life for cancer patients [6]. However, new anticancer drugs usually have extremely high prices, making them inaccessible for patients in economically developing countries [7,8] and challenging the affordability and sustainability of drug programmes worldwide [9]. The huge cumulative medical expenditure related to oncological treatment further causes financial toxicity, referring to the objective financial burden and subjective financial distress of patients and society, causing significant concern [10].

To decrease oncology treatment costs and make novel anticancer drugs more affordable, China has implemented a series of policies, incorporating drug regulatory reform, national drug price negotiation, and reimbursement lists. Seven rounds of such negotiations have been conducted by 2023. A pilot price negotiation was performed in 2016 and afterwards formally employed as a criterion for inclusion in the National Reimbursement Drug List (NRDL) since 2017. Simultaneously, the Ministry of Human Resources and Social Security adopted a pharmacoeconomic evaluation as a measuring tool for medicine listing and pricing negotiation [11]. The average price discount for innovative drugs surpassed 50% after market entry [12]. According to prior studies, lung cancer (LC) and breast cancer (BC) were the most common neoplastic indications included in NRDL [13,14], and the ones showing the highest incidence among males and females in China, respectively [1,4]. Thus, we aimed to study the value of price-negotiated anticancer drugs targeted to LC and BC.

Value-based health care is defined as maximising health outcomes achieved per dollar spent [15]. Due to its growing popularity, emphasis has been placed on whether the high-priced new anticancer drugs are commensurate with related survival benefits. In clinical trials, regulatory approvals of new cancer drugs are supported by overall survival (OS), progression-free survival (PFS), or objective response rate (ORR) [16]. OS is recognised as a reliable endpoint, while PFS or ORR are used as a surrogate endpoint [14,16]. However, most cancer drugs approved in China lack the evidence of OS benefit. To better quantify their clinical benefit, the American Society of Clinical Oncology Value Framework (ASCO-VF) [17,18] and the European Society for Medical Oncology Magnitude of Clinical Benefit Scale (ESMO-MCBS) [19] have been developed as evaluation frameworks to analyse efficacy, toxicity, and quality of life of cancer therapies.

Several similar studies were conducted to estimate the prices and clinical benefits of new anticancer drugs in China and elsewhere, either based on traditional clinical endpoints or on aforementioned value frameworks. Most results indicated that anticancer costs were independent or weakly associated with improvement in clinical benefits [7,13,20-23], while few studies had converse findings [24]. However, little attention has been paid to the relation between clinical value and costs on specific cancer sites before and after the implementation of drug price negotiation. Simultaneously, no research identified the “value” with composition of typical clinical endpoints and the quantitative results of ASCO-VF and ESMO-MCBS in China. We therefore compared the correlation between monthly costs and clinical benefits of new anticancer drugs before and after entering NRDL through the price negotiation for LC and BC in China. We hypothesised that the value of innovative anticancer drugs was not positively correlated with their cycle costs before the price negotiation, and that the price negotiation generated better alignment of cycle costs and value due to the supporting of economic evaluation, meaning the benefits of innovative anticancer drugs were positively in line with incremental costs after the price negotiation.

## METHODS

### Data sources and sample identification

We performed a retrospective study in which we identified all innovative anticancer drugs for LC and BC between 2016 and 2023 under publicly available NRDL announced by the National Healthcare Security Administration (NHSA) [25-29]. We searched PubMed, Embase, and Web of Science with the search terms “((lung cancer) OR (breast cancer)) AND ((drug name) OR (NCT number)) AND (clinical trial OR randomized controlled trial)”, published from 1 May 2016 to 1 June 2023. As some key clinical trials were not published in peer-reviewed literature, we included randomised controlled trials (RCTs) registered on the clinicaltrials.gov website or noted on drug instructions [30]. One researcher (RM) collected and integrated the entitled new anticancer drugs, while two others (YB and YL) manually searched through databases, website, and instructions. They then extracted the median OS and PFS data from critical clinical trials, resolving discrepancies with a senior reviewer (XL).

We included RCTs in LC and BC and studies with available data regarding OS and/or PFS, and excluded studies with agents only for adjuvant or neo-adjuvant therapy, cancer supportive care, phase I/II/IV, and single-arm trials, those with OS and/or PFS data that were not fully achieved, and those with non-Chinese or non-East Asian populations or with <25% Chinese patients in the multi-centre clinical trials. If a new therapeutic drug had multiple disclosed trials and more than two of them met the inclusion criteria, we featured those with the optimal treatment outcomes. This study was exempt from ethical procedures as it was based on published literature and universal data without involving patient information.

### Monthly costs calculation

We estimated price data of anticancer therapeutic regimens (LC and BC) for novel drugs outside of NRDL or within NRDL from the winning bid prices provided by the government [31], which is the price purchased by medical institutions like hospitals and health centres. However, some negotiated anticancer drugs have separate medical insurance payment, general term of price, quantity, and compensation proportion standards for drugs in China after price negotiation, so their cost calculation inclined to payment standards. Regardless of price changes after renewal, we measured only the initial price of each anticancer drug enrolled in NRDL.

We calculated the monthly treatment costs for each eligible drug over an average of 28 days based on the prescription and dosing information on the latest labels and drug instructions approved by the National Medical Products Administration (NMPA). The dosage was set at a body surface area of 1.72m^2^ with an average adult weighing 65kg [32]. If the initial dose was inconsistent with the subsequent one, we then regarded the average dose of each cycle as the standard. All monthly costs of medication regimens were eventually adjusted to four-week period price. When there were several approved doses (e.g. 50 mg per tablet and 100 mg per tablet), we chose the dosage with the lowest costs per unit. All expense data were converted into US$ at the average exchange rate of the first half of 2023 (CNY1 = US$0.14455) [33].

### Clinical outcome extraction and translation

We extracted the median OS and PFS of the experimental and control groups from key clinical trials. We calculated OS and PFS gains by the percentage difference in median OS and PFS between the two regimens as the traditional measure of clinical benefits, expressed by incremental percentage of OS (∆OS%) and incremental percentage of PFS (∆PFS%), respectively. We then defined outcomes that exceed the 75^th^ percentile of ∆OS% and ∆PFS% as valid benefits per the following formula:

∆*OS%* = *median OS* (*months*) *in experimental group* − *median OS* (*months*) *in control group*/*median OS* (*months*) *in control group*

∆*PFS%* = *median PFS* (*months*) *in experimental group* − *median PFS* (*months*) *in control group*/*median PFS* (*months*) *in control group*

We converted the extracted OS and PFS into intuitive efficacy scores by using two established value frameworks proposed by ASCO and ESMO, to reinforce the comparison between clinical benefits and monthly costs. The ASCO-VF version 2.0 was a hundred-point scale, which was divided into two evaluation forms based on cancer progression, including the therapeutic benefit, toxicity, bonus points, and net health benefit [17,18,34]. The scores of ESMO-MCBS, version 1.1 ranged from 1 to 5. It assessed the type of study, clinical endpoints, and benefit of the control drug. A grade of A-B in curative settings or 4-5 in palliative settings on the ESMO-MCBS was defined as meaningful clinical benefit, while the score of ASCO-VF lacked the obvious definition of “meaningful value” [19,34]. Here we set the 75^th^ percentile value as the cutoff, which we considered to be an effective score if it was greater than the critical value.

### Statistical analysis

As some continuous variables for the correlation analysis were not normally distributed per the Shapiro-Wilk test, we used the Spearman’s rank correlation coefficient (r_s_) to test the correlations between monthly drug costs and clinical benefit in LC and BC or lines of therapy. We visualised the outcomes as scatter plots or box plots. We checked for consistency between ASCO-VF and ESMO-MCBS, ∆OS% and ∆PFS% by Cohen’s kappa statistics (κ); the κ value varies from 0 to 1, with higher values indicating higher agreement. We generated plots in Microsoft Excel 2021 (Microsoft Inc, Seattle WA, USA) and used SPSS, version 23.0 (IBM Corp., Armonk, NY, USA) for correlation analysis, coefficient estimation, and consistency test. We considered *P*-values <0.05 as statistically significant.

## RESULTS

### Features of innovative anticancer drugs

**Figure 1:**
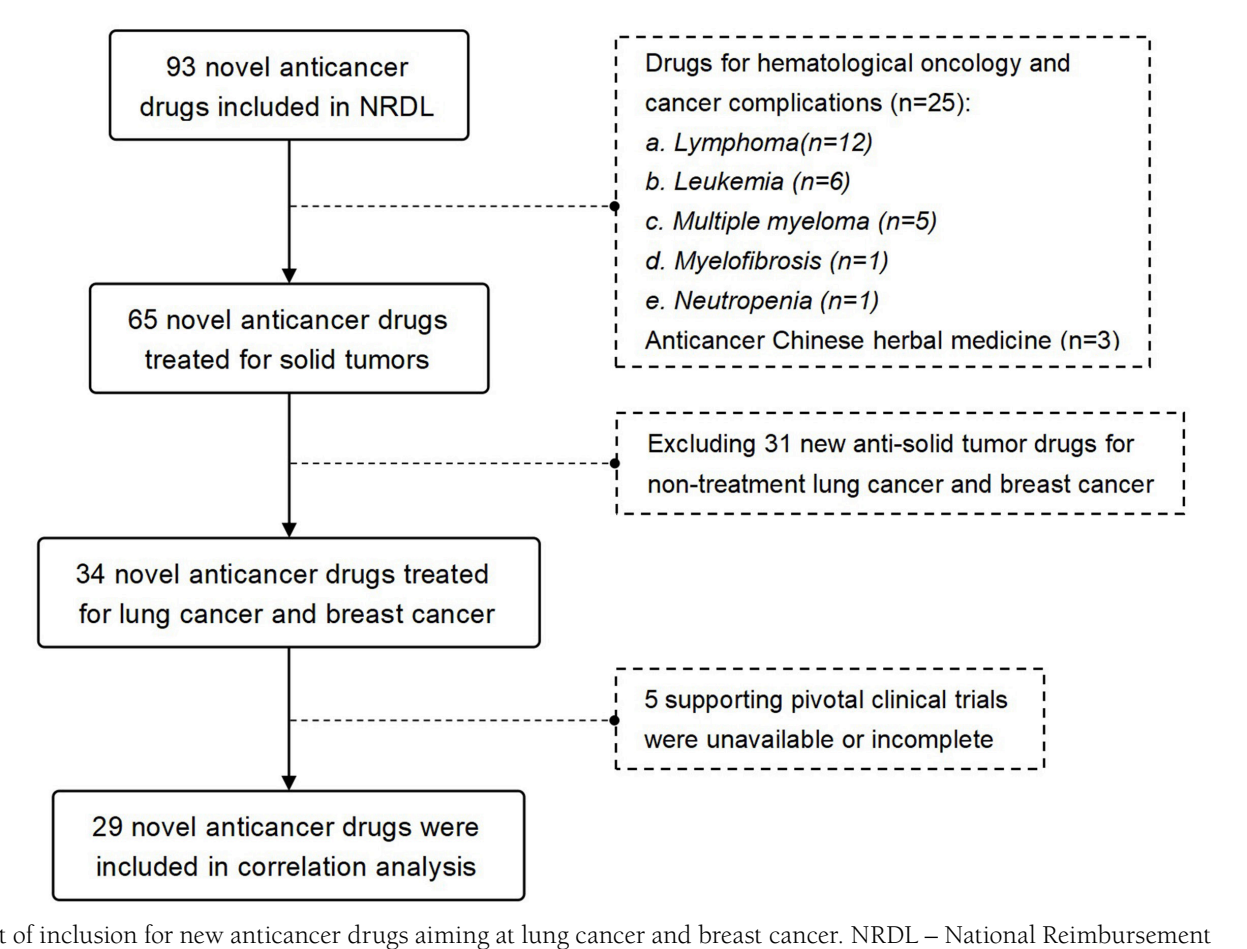
Flowchart of inclusion for new anticancer drugs aiming at lung cancer and breast cancer. NRDL – National Reimbursement Drug List.

Ninety-three novel anticancer drugs had been negotiated and included in NRDL between May 2016 and June 2023, among which 65 (69.89%) were treatments for solid tumours and 25 were approved for treating hematologic cancers, including three Chinese herbal medicines. Among solid tumour anticancer drugs, 21 (32.31%) and 13 (20.00%) were treated non-small cell lung cancer (NSCLC) and BC, respectively. Based on the result of database retrieval, five drugs lacked key clinical trials (two for NSCLC and three for BC). We thus included 29 drugs with key trial data for subsequent analyses, 18 of which were conducted outside of China, while 11 were domestic, with 21 first-line and nine second-line drugs (**Table 1** and **Figure 1**).

**Table 1:** Characteristics of new anticancer drugs for lung cancer and breast cancer in China

Generic name	Indication	Year of initial NRDL price negotiation	Origin	Lines of therapy	Evaluation index	ASCO-VF scores	ESMO-MCBS scores
Icotinib	EGFR mutation-positive locally advanced or metastatic NSCLC	2016	Domestic	First-line	PFS	53.02	4
Icotinib	EGFR mutation-positive locally advanced or metastatic NSCLC	2016	Domestic	First-line	OS	4.38	1
Gefitinib	EGFR mutation-positive locally advanced or metastatic NSCLC	2016	Imported	Second-line	OS	-5.12	2
Erlotinib	EGFR mutation-positive advanced NSCLC	2017	Imported	First-line	PFS	67.51	4
Bevacizumab	Advanced non-squamous NSCLC	2017	Imported	First-line	OS	29.24	4
Lapatinib	HER2 positive advanced or metastatic BC	2017	Imported	Second-line	OS	32.90	3
Crizotinib	ROS1 mutation-positive advanced NSCLC; ALK mutation-positive locally advanced or metastatic NSCLC	2018	Imported	First-line	OS	26.66	2
Crizotinib	ROS1 mutation-positive advanced NSCLC; ALK mutation-positive locally advanced or metastatic NSCLC	2018	Imported	Second-line	PFS	58.47	4
Osimertinib	EGFR T790M mutation-positive locally advanced or metastatic NSCLC	2018	Imported	First-line	OS	20.29	3
Ceritinib	Prior receiving crizotinib or intolerant to crizotinib with ALK mutation-positive locally advanced or metastatic NSCLC	2018	Imported	First-line	PFS	55.62	4
Anlotinib	Locally advanced or metastatic NSCLC after receiving systemic chemotherapy	2018	Domestic	Second-line	OS	41.33	4
Afatinib	EGFR mutation-positive locally advanced or metastatic NSCLC	2018	Imported	First-line	PFS	47.77	4
Afatinib	Locally advanced or metastatic squamous NSCLC after platinum-containing chemotherapy	2018	Imported	Second-line	OS	32.33	2
Alectinib	ALK mutation-positive locally advanced or metastatic NSCLC	2019	Imported	First-line	OS	60.48	5
Pertuzumab	HER2 positive locally advanced BC or neoadjuvant for early BC	2019	Imported	First-line	OS	49.18	4
Pyrotinib	HER2 positive advanced or metastatic BC	2019	Domestic	Second-line	PFS	48.96	3
Almonertinib	EGFR T790M mutation-positive advanced or metastatic NSCLC	2020	Domestic	First-line	PFS	40.98	3
Camrelizumab	EGFR mutation-negative and ALK mutation-negative NSCLC	2020	Domestic	First-line	OS	37.26	4
Dacomitinib	EGFR exon 19 deletion-mutation or exon 21 L858R substitution-mutation locally advanced or metastatic NSCLC	2021	Imported	First-line	OS	41.86	4
Furmonertinib	EGFR T790M mutation-positive locally advanced or metastatic NSCLC	2021	Domestic	First-line	PFS	54.55	3
Tislelizumab	Unresectable locally advanced or metastatic squamous NSCLC; EGFR mutation-negative or ALK mutation-negative or unresectable non-squamous NSCLC	2021	Domestic	First-line	PFS	52.69	3
Sintilimab	Unresectable locally advanced or recurrent squamous NSCLC; EGFR mutation-negative or ALK mutation-negative non-squamous NSCLC	2021	Domestic	First-line	OS	52.29	4
Ensartinib	ALK mutation-positive locally advanced or metastatic NSCLC	2021	Domestic	First-line	PFS	32.97	3
Neratinib	HER2 positive early BC	2021	Imported	Second-line	OS	22.09	4
Eribulin	HER2 positive advanced or metastatic BC	2021	Imported	Second-line	OS	9.18	1
Abemaciclib	HR positive or HER2 negative advanced or metastatic BC	2021	Imported	Second-line	OS	36.78	4
Brigatinib	ALK mutation-positive locally advanced or metastatic NSCLC	2023	Imported	First-line	PFS	71	4
Lorlatinib	ALK mutation-positive locally advanced or metastatic NSCLC	2023	Imported	First-line	PFS	69.48	4
Palbciclib	HR positive or HER2 negative advanced or metastatic BC	2023	Imported	First-line	OS	24.20	3
Utidelone	Recurrent or metastatic BC after receiving at least one chemotherapy	2023	Domestic	First-line	OS	38.85	3
Ado-trastuzumab emtansine(T-DM1)	HER2-positive early BC	2023	Imported	First-line	OS	31.64	3
Dalpiciclib	HR positive or HER2 negative advanced or metastatic BC	2023	Domestic	First-line	PFS	21.42	3
Dalpiciclib	HR positive or HER2 negative advanced or metastatic BC	2023	Domestic	First-line	OS	60.58	4

NRDL – National Reimbursement Drug List, ASCO-VF – American Society of Clinical Oncology Value Framework, ESMO-MCBS – European Society for Medical Oncology Magnitude of Clinical Benefit Scale, NSCLC – non-small lung cancer, BC – breast cancer, EGFR – epidermal growth factor receptor, ROS1 – Recombinant C-Ros Oncogene 1 Receptor Tyrosine Kinase, ALK – anaplastic lymphoma kinase, HR – hormone receptor, HER2 – human epidermal growth factor receptor-2

The median monthly treatment cost of 29 new drugs before NRDL was US$3381.31 (interquartile range (IQR) = 2243.40-5327.51), ranging from US$1678.29 to US$10118.50, with a six-fold price divergence. After entering NRDL, the median cost per month was US$1095.88 (IQR = 799.08-1644.26), which was about three-tenths of the previous median cost. The median monthly costs for NSCLC were US$3967.40 and US$1204.97 before and after price negotiation, while the median monthly costs for BC were US$2246.31 and US$898.52, respectively.

### Overview of clinical outcomes

All of the 29 included drugs had mature PFS outcomes, while 22 had OS outcomes. Specifically, 14 of 19 anti-LC agents tracked OS data and eight out of 10 anti-BC drugs had mature OS outcomes available. The ∆OS% ranged from -4.98% to 52.38%, while ∆PFS% fluctuated from 0% to 285.71%. The median ∆OS% of anti-LC and anti-BC agents was 22.24% (IQR = 6.45-29.47), while their median ∆PFS% was 83.82% (IQR = 50.41-104.05), respectively. We set the 75^th^ percentile of ∆OS% (29.47%) and ∆PFS% (104.05%) as boundary. The results were generally consistent with the selected cancers separately. ∆OS% was 36.40% and ∆PFS% was 107.76% in NSCLC, while ∆OS% was 26.75% and ∆PFS% was 97.66% in BC. Regarding therapy lines, the ∆OS% and ∆PFS% were 23.43% and 81.65% for first-line and 15.59% and 83.82% for second-line drugs, respectively.

The scores for the evaluation of ASCO-VF ranged from -5.14 to 71.00, with a median ASCO-VF score 40.98 (IQR = 27.95-53.79) for all drugs, 44.82 for NSCLC drugs, and 32.90 for BC drugs. We likewise set a 75^th^ percentile score of 53.79 as the cutoff value to identify “meaningful benefit”; 25 drugs were under the threshold, while nine were above. As for ESMO-MCBS, 17 drugs scored above the “high benefit” (4-5) defined in this framework, and 17 scored below the cutoff. Furthermore, the median score of first-line agents was estimated at 44.82, while the median of second-line drugs was 32.90. A sum of 13 first-line drugs was exceeding the “high value” of ESMO-MCBS and four second-line drugs went beyond the dividing value.

### Correlation between monthly costs and OS/PFS gain

**Figure 2:**
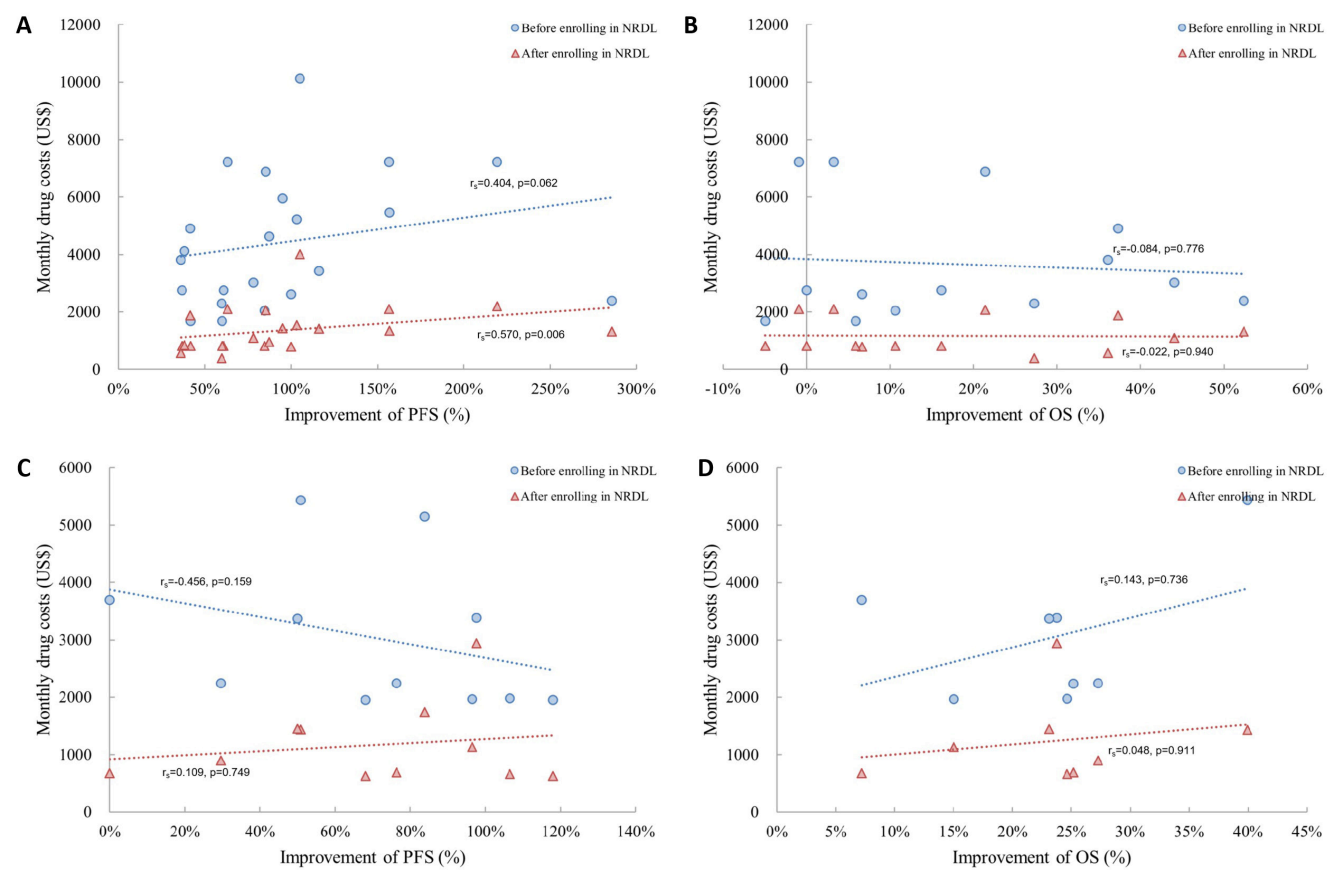
Correlation between monthly costs and percentage improvement among specific cancers. **Panel A.** Value assessment of ΔPFS% in NSCLC. **Panel B.** Value assessment of ΔOS% in NSCLC. **Panel C.** Value assessment of ΔPFS% in BC. **Panel D.** Value assessment of ΔOS% in BC. The blue scatter indicated that drugs were out of NRDL while the red scatter indicated that drugs were within NRDL. ΔPFS% – percentage improvement of progression-free survival, ΔOS% – percentage improvement of overall survival, NSCLC – non-small lung cancer, BC – breast cancer, NRDL – National Reimbursement Drug List.

The incremental improvement of anticancer drugs and their costs were basically irrelevant (*P* > 0.05). However, the incremental PFS gains (∆PFS%) of new drugs included in the reimbursement list (r_s_ = 0.353; *P* = 0.044) showed a weak positive relationship with the average monthly costs. We found none or only a weak correlation between either OS or PFS gain and monthly costs regardless of enrolment when analysing medication for LC or BC separately, comparable to the overall analysis of new anticancer drugs for two cancers (**Figure 2**).

When only analysing the lines of treatment, the incremental gains of PFS and OS in first-line or second-line therapy was not significantly correlated with cost per month before and after being included in NRDL (*P* > 0.05) (**Figure S1** in the **Online Supplementary Document**).

### Association between monthly costs and framework scores

**Figure 3:**
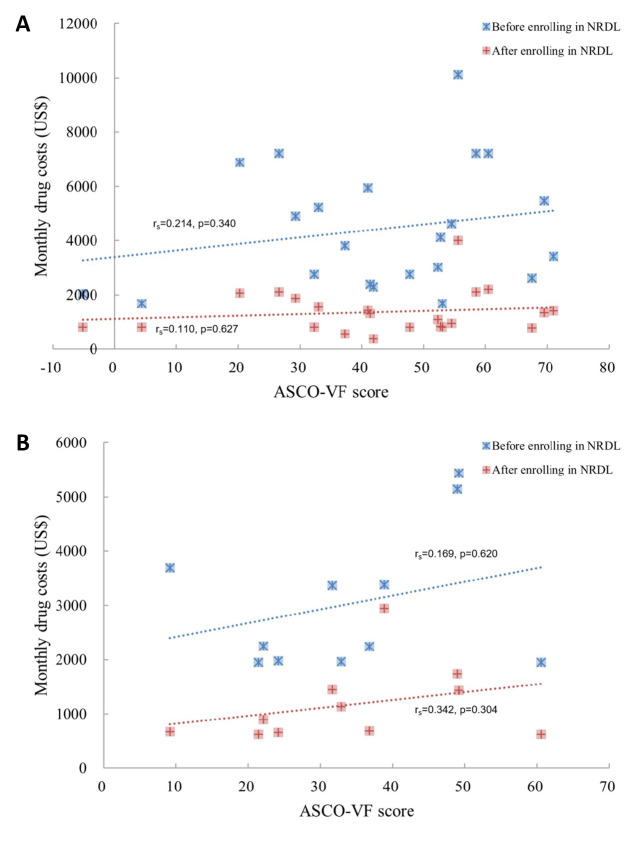
Correlation between monthly costs and ASCO-VF scores among specific cancers**. Panel A.** Value assessment of ASCO-VF scores in NSCLC; **Panel B.** Value assessment of ASCO-VF scores in BC. The blue scatter indicated that drugs were out of NRDL while the red scatter indicated that drugs were within NRDL. ASCO-VF – American Society of Clinical Oncology Value Framework, NSCLC – non-small lung cancer, BC – breast cancer, NRDL – National Reimbursement Drug List.

**Figure 4:**
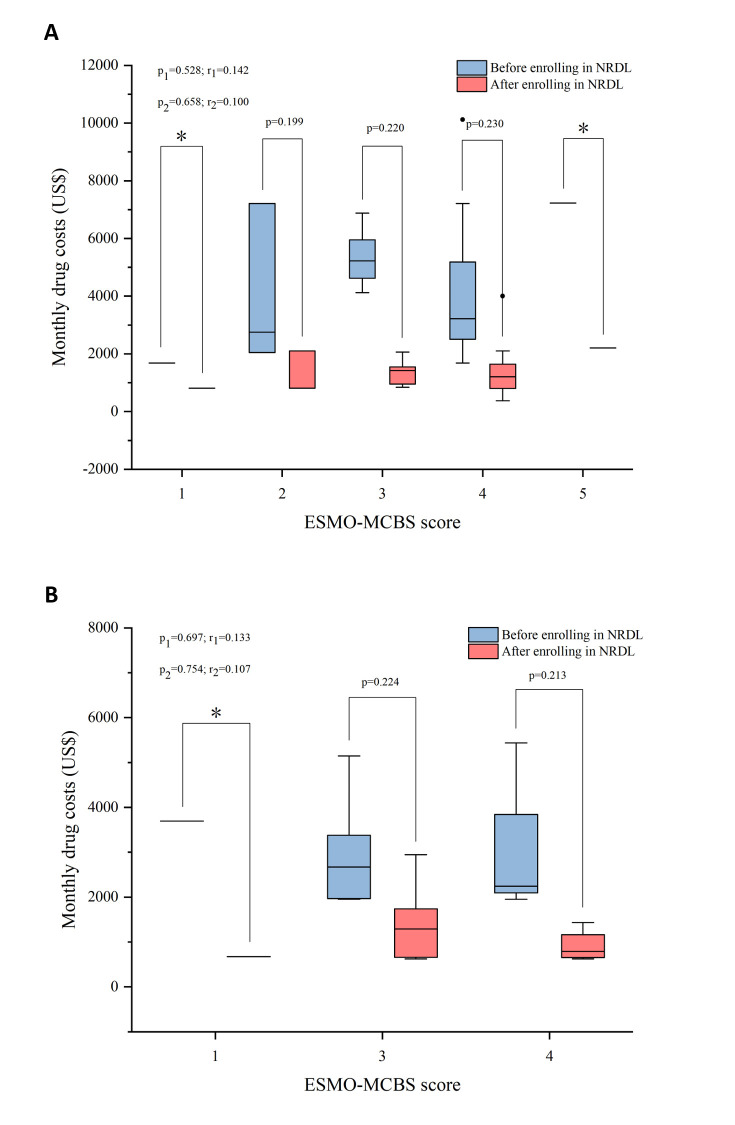
Correlation between monthly costs and ESMO-MCBS scores among specific cancers. **Panel A.** Value assessment of ESMO-MCBS scores in NSCLC. **Panel B.** Value assessment of ESMO-MCBS scores in BC. The blue box plot indicated that drugs were out of NRDL while the red box plot indicated that drugs were within NRDL. p_1_, p_2_ values separately represented the significance of the correlation between ESCO-MCBS scores and monthly costs before and after inclusion in NRDL while r_1_, r_2_ separately represented the corresponding spearman’s correlation coefficient. p value was the significance of the correlation between each group after being stratified. *Sample size is too small to be compared after stratification. ESMO-MCBS – European Society for Medical Oncology Magnitude of Clinical Benefit Scale, NSCLC – non-small lung cancer, BC – breast cancer, NRDL – National Reimbursement Drug List.

We used ASCO-VF and ESMO-MCBS to quantify the clinical outcomes for in-depth analysis. We did not observe significant associations between monthly drug costs and the scores of ASCO-VF or the scores of ESMO-MCBS before and after NRDL admissions, in line with the results of framework scores and costs in LC and BC, respectively (*P* > 0.05) (**Figure 3** and **Figure 4**). Stratified by lines of therapy, both first-line and second-line drug treatments showed that the clinical value had nothing to do with the costs (*P* > 0.05) (Figure S2 in the **Online Supplementary Document**).

### Agreement between ASCO and ESMO, incremental OS and PFS gains

Clinical outcomes were displayed by ∆OS% and ∆PFS%, together with quantitative scores of ASCO-VF and ESMO-MCBS. It showed a moderate agreement between ASCO-VF and ESMO-MCBS, with a κ estimated at 0.429 (*P* = 0.001). However, the agreement between ∆OS% and ∆PFS% was not clear (κ = 0.049; *P* = 0.801). ASCO-VF and ∆PFS% showed a relatively strong consistency (κ = 0.542; *P* = 0.002), while the consistency between ASCO-VF and ∆OS% could not be determined (κ = -0.222; *P* = 0.254). Agreement between ESMO-MCBS and ∆OS% (κ = 0.353; *P* < 0.05), ESMO-MCBS and ∆PFS% (κ = 0.206; *P* < 0.05) were only fair. We found similar results after sub-analyses by cancer types and therapy lines (*P* < 0.05).

## DISCUSSION

To our knowledge, this is the first study in China to explore the association between drug cost and the value of specific cancers, considering the implementation of drug negotiation. We examined 29 new anticancer drugs for LC or BC that were listed in NRDL after national price negotiations from 2016 to 2023, using both the incremental percentage of OS and PFS and the quantitative scores of ASCO-VF and ESMO-MCBS to comprehensively assess their clinical value. We thus confirmed our first, but not our second hypothesis, even though drug prices were reduced overall after negotiation. This suggest clinical benefits of innovative anticancer drugs were generally not significantly or just weakly associated with costs for treatment after controlling for the specific cancer sites and the lines of therapy.

Previously, two studies based on the OS and PFS conducted in China and Japan found a lack of correlation between anticancer drug costs and their benefits [7,22]. The previous Chinese study focused the price negotiation policy. However, there is a lack of drug price negotiations between the drug manufacturers and the government in Japan. The findings of both studies were generally consistent with ours. We can observe that, concerning the magnitude of OS and PFS gains, high prices might not explicitly yield the equivalent benefit regardless of the implementation of negotiation policy. In fact, to accelerate the launch and access to drugs of treatment shortage in the reimbursement list, policymakers put an increasing emphasis on the clinical consequences or cost-effectiveness of drugs rather than the quality of evidence [35], resulting in almost a quarter of launched drugs to be approved based on surrogate endpoints rather than the most reliable OS outcome [14]. Other countries were confronted with the same dilemma [36,37]. Consequently, new drugs, especially new anticancer medicines with low or uncertain clinical benefits, might be prioritised for price negotiations, exacerbating the disease burden on patients and lowering the accessibility of superior medical resources.

Despite clinical endpoints being commonly employed to measure clinical efficacy, multiple frameworks have been proposed to reflect the clinical benefits, with ASCO-VF and ESMO-MCBS being applied most frequently. These frameworks had different conceptual definitions of “value” along with divergent scoring systems; however, the results of their value assessment were unified, demonstrating convergent validity and inter-rater reliability [13]. Based on these frameworks, previous studies also suggested that drug costs were either unrelated or had only weak associations with treatment outcomes, both in China and most other countries with or without negotiation policy [13,20,21,38], which is in line with our findings. This could be a consequence of the interplay between price negotiation and profit-oriented enterprises or patient’s benefit-driven government. The innovative anticancer drugs were listed by pharmaceutical enterprises at high prices under the patent protection in the early stage. Although the price decline was considerable after negotiation, the price remained high compared with other drugs in the reimbursement list. However, the negotiated decrease in the price of new agents was closely related to their innovative nature. If the drug was unique within the scope of the indication, it may be accepted at a relatively high rate despite the limited improvement in clinical efficacy. Conversely, if there were alternatives with the same indication, the chance of being accepted was relatively low, or it would not share the same discount as the previously existing one.

Recently, the national negotiation procedure was gradually institutionalised and several other influential reimbursement policies were introduced and implemented successively in China, such as priority and conditional approvals, dynamic updates of NRDL, pharmacoeconomic analyses for price estimation, and others [39]. The national reimbursement policy, which took drug price negotiation and dynamic updates of NRDL as the main measure, allowed for advantages of national purchase and price-for-quantity. Pharmacoeconomic evidence is increasingly used as the basis for drugs to be included in NRDL. During the price negotiations between NHSA and the pharmaceutical manufacturer, the bidding for price of manufacturer could not exceed 15% of the floor price provided by the NHSA, else the drug would be rejected [40]. The floor price was based on the pharmacoeconomic evaluation (cost-effectiveness analysis and budget impact analysis), which could pose positive or negative impacts on the correlations between drug price and its value [40].

In our study, the expenditure of novel anticancer drugs in China has dropped after being listed in the NRDL, but its core pricing still did not meet the value-based healthcare. Despite the cost-effectiveness analysis and health technology assessment (HTA) were increasingly working as supporting evidence in policy implementation such as price negotiation, their potential did not fully give way to exploitation. This could be related to the transparency of pharmacoeconomic evidence in China. Thus, the price negotiation did not strengthen the association between drug costs and benefits positively, which could serve as a lesson for other countries conducting their own negotiation policy. Pharmacoeconomics or HTA needs to be more deeply and institutionally applied to the government decision-making process. As with the National Institute for Health and Care Excellence (NICE), countries should try to establish an independent HTA and pharmacoeconomic assessment agency with national characteristics, neither restricted by the corporate nor government, to provide more neutral and transparent supporting evidence. Additionally, authorities should adopt a comprehensive and dynamic evaluation rule to re-assess the clinical and economic value of new medicines concerning real world data within two to five years after market access.

This study has several limitations. First, OS is recognised as a valid clinical endpoint for drug approval, but due to its novelty in several clinical trials, our framework points were partly based on the PFS as a surrogate benefit. Furthermore, we only obtained phase III RCTs existing as of 2023. Updated clinical data and post-approval clinical studies would lead to changes on efficacy over time, which would cause biases in subsequent analysis. Second, we only used data from oncology trials in the Chinese and Asian populations or multi-centre RCTs with a ≥25% proportion of the Chinese population. Moreover, some of the agents were only approved by NMPA and not be available in other countries, making them less generalisable. Third, we only analysed data on LC and BC, which have a high incidence and a significant proportion of drugs included in NRDL, and thus cannot conclude whether other solid tumours would have similar results. Finally, factors affecting the value of innovative drugs were multi-dimensional, while our assessment counted on disclosed trials, only taking the clinical benefit and cost into consideration, necessitating future studies.

## CONCLUSIONS

We found no explicit correlation between therapeutic benefit and monthly costs of price-negotiated anticancer drugs in NSCLC and BC, irrespective of whether we used OS and PFS gain as clinical evaluation or whether we applied the value frameworks as the visualisation of clinical benefits before and after entering NRDL. Price negotiation should adhere to the concept of value-based health care. However, more in-depth studies are needed to confirm our findings, and more credible or transparent negotiation evidence with comprehensive value assessment should be provided to facilitate value-based antineoplastic agents and healthcare allocation.

## Additional material


Online Supplementary Document
