# Supplementary material for: The correlation between the costs and clinical benefits of national price-negotiated anticancer drugs for specific cancers in China
Source: J Glob Health. 2023 Nov 9;13:04140. doi: 10.7189/jogh.13.04140 (PMC10629928; doi:10.7189/jogh.13.04140)
Supplement: Online Supplementary Document [file jogh-13-04140-s001.pdf]

ONLINE SUPPLEMENTARY DOCUMENT

Title: The correlation between the costs and clinical benefits of national price-negotiated anticancer drugs for specific cancers in China

Authors: Yuwen Bao[1], Yanyan Liu[1,2], Rui Ma[3], Pei Zhang[3], Xin Li[1,3,4]

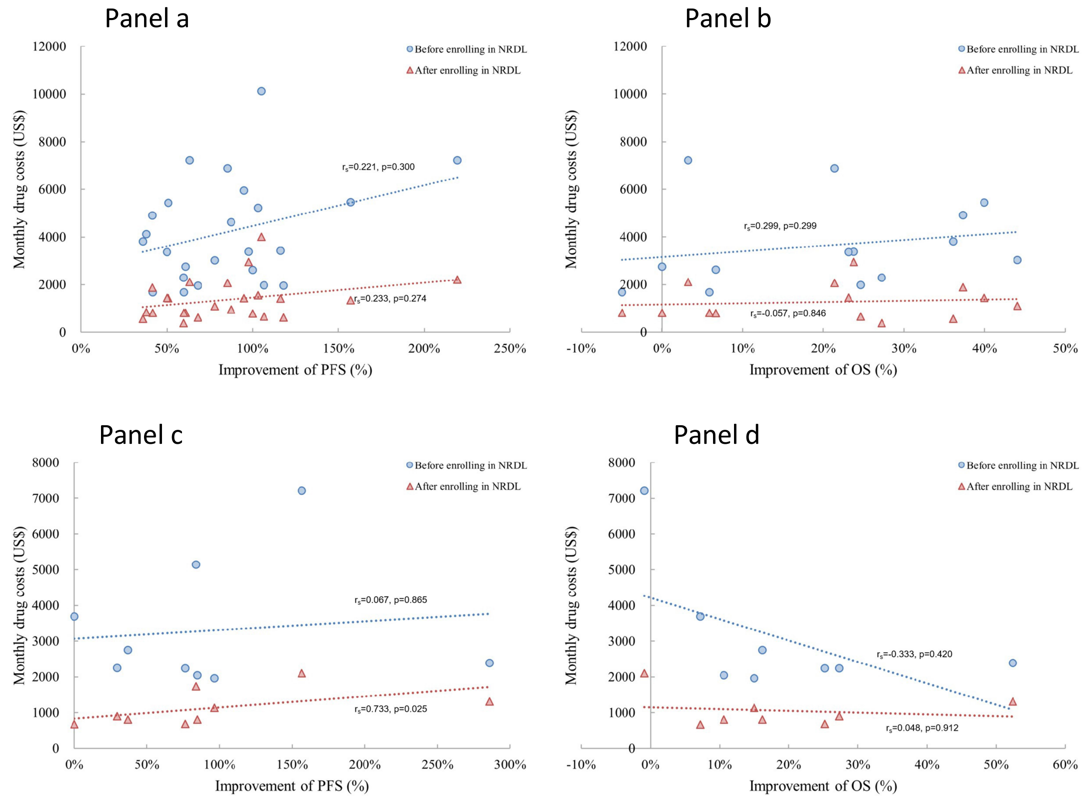

Figure S1 Correlation between monthly costs and percentage improvement among different lines of therapy. Panel a: value assessment of ΔPFS% in first-line therapy; Panel b: value assessment of ΔOS% in first-line therapy; Panel c: value assessment of ΔPFS% in second-line therapy; Panel d: value assessment of ΔOS% in second-line therapy. The blue scatter indicated that drugs were out of NRDL while the red scatter indicated that drugs were within NRDL. ΔPFS%, percentage improvement of progression-free survival; ΔOS%, percentage improvement of overall survival; NRDL, National Reimbursement Drug List.

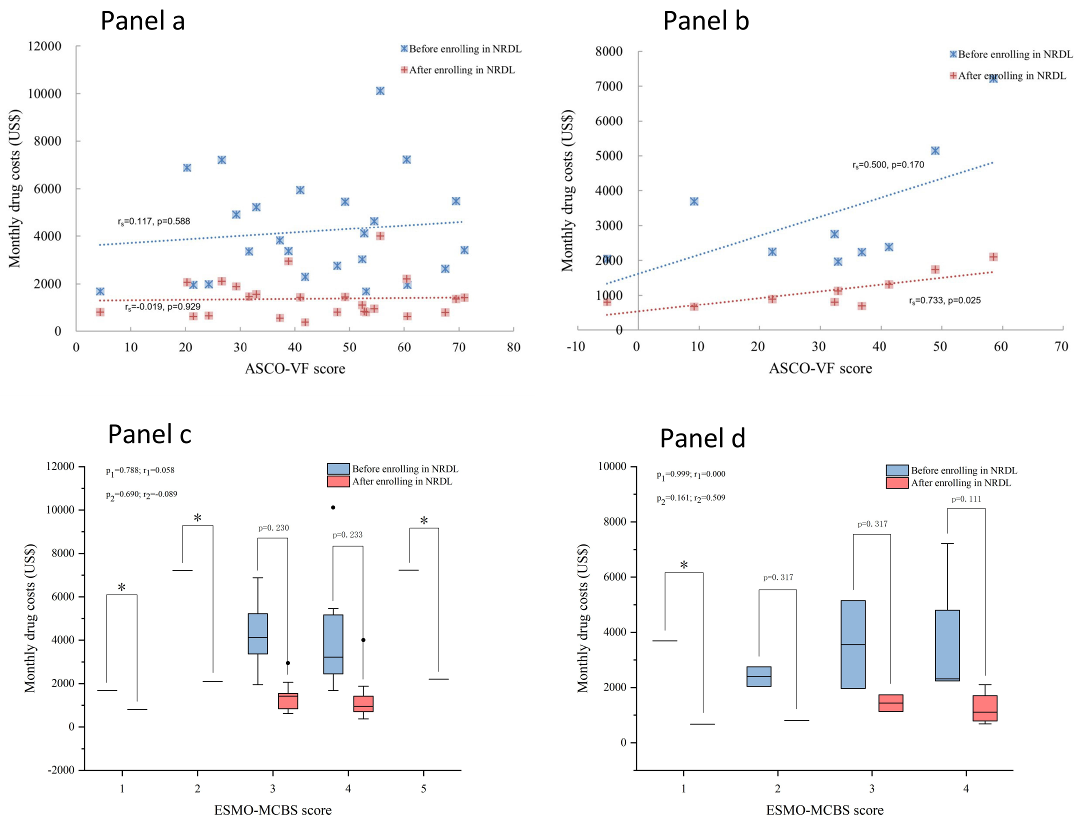

Figure S2 Correlation between monthly costs and value frameworks' scores among different lines of therapy. Panel a: value assessment of ASCO-VF scores in first-line therapy; Panel b: value assessment of ASCO-VF scores in second-line therapy; Panel c: value assessment of ESMO-MCBS scores in first-line therapy; Panel d: ESMO-MCBS scores in second-line therapy. The blue scatter/box plot indicated that drugs were out of NRDL while the red scatter/box plot indicated that drugs were within NRDL. p_1, p_2 values separately represented the significance of the correlation between ESCO-MCBS scores and monthly costs before and after inclusion in NRDL while r_1, r_2 separately represented the corresponding spearman's correlation coefficient. p value was the significance of the correlation between each group after being stratified. * means that the simple size is too small to be compared after stratification. ASCO-VF, American Society of Clinical Oncology Value Framework; ESMO-MCBS, European Society for Medical Oncology Magnitude of Clinical Benefit Scale; NSCLC, non-small lung cancer; BC, breast cancer; NRDL, National Reimbursement Drug List.
